# Singularity of the time-energy uncertainty in adiabatic perturbation and cycloids on a Bloch sphere

**DOI:** 10.1038/srep20824

**Published:** 2016-02-26

**Authors:** Sangchul Oh, Xuedong Hu, Franco Nori, Sabre Kais

**Affiliations:** 1 Qatar Environment and Energy Research Institute, Hamad Bin Khalifa University, Qatar Foundation, PO Box 5825, Doha, Qatar; 2 Department of Physics, University at Buffalo, State University of New York, Buffalo, New York 14260-1500, USA; 3 Center for Emergent Matter Science, RIKEN, Saitama 351-0198, Japan; 4 Physics Department, The University of Michigan, Ann Arbor, Michigan, 48109-1040, USA; 5 Department of Chemistry, Department of Physics and Birck Nanotechnology Center, Purdue University, West Lafayette, IN 47907 USA

## Abstract

Adiabatic perturbation is shown to be singular from the exact solution of a spin-1/2 particle in a uniformly rotating magnetic field. Due to a non-adiabatic effect, its quantum trajectory on a Bloch sphere is a cycloid traced by a circle rolling along an adiabatic path. As the magnetic field rotates more and more slowly, the time-energy uncertainty, proportional to the length of the quantum trajectory, calculated by the exact solution is entirely different from the one obtained by the adiabatic path traced by the instantaneous eigenstate. However, the non-adiabatic Aharonov- Anandan geometric phase, measured by the area enclosed by the exact path, approaches smoothly the adiabatic Berry phase, proportional to the area enclosed by the adiabatic path. The singular limit of the time-energy uncertainty and the regular limit of the geometric phase are associated with the arc length and arc area of the cycloid on a Bloch sphere, respectively. Prolate and curtate cycloids are also traced by different initial states outside and inside of the rolling circle, respectively. The axis trajectory of the rolling circle, parallel to the adiabatic path, is shown to be an example of transitionless driving. The non-adiabatic resonance is visualized by the number of cycloid arcs.

Perturbation theory^1^,^2^ is widely used in many fields of science and engineering as an effective method to find an approximate solution to a given problem, expressed in terms of a power series in a small parameter. In regular perturbation calculations, one only keeps the first few terms of the expansion to obtain a good approximate solution to the exact one, as the small parameter goes to zero. However, there are many interesting problems that have no such uniform asymptotic expansion. These involve singular perturbations^2^,^3^,^4^,^5^,^6^,^7^. A classic example of this singular perturbation is the flow in the limit of zero viscosity^8^. When the viscosity, a small parameter, approaches zero, the solution of the Navier-Stokes equation gives a completely different solution from the one obtained by taking zero viscosity from the beginning.

Adiabatic perturbation^1^ is one of the fundamental approximations used in many fields. Its classic applications include the Born-Oppenheimer approximation^9^ of decoupling the fast electronic motion from the slow ionic one, and adiabatic quantum computation^10^, an alternative to the quantum circuit model for quantum computing. The adiabatic theorem dictates that as long as a system changes slowly enough, a quantum system starting from an eigenstate would remain in the instantaneous eigenstate of the time-dependent Hamiltonian up to the dynamical and Berry phases^11^,^12^. It may seem quite reasonable then that all physical properties in the adiabatic limit should be obtainable from the instantaneous eigenstate. However, this conjecture has never been proved.

In this paper, we reveal singular features of the adiabatic approximation by studying the quantum dynamics of a spin-1/2 particle in a uniformly rotating magnetic field. Its quantum trajectory is shown to be a cycloid on the Bloch sphere, traced by a point on a rolling circle, with a radius determined by the angular speed of the magnetic field, along the adiabatic path of the instantaneous eigenstate. We find the two basic geometric quantities, the length and the enclosed area of the quantum trajectory, approach different limits in the adiabatic limit. As the rotation of the magnetic field is slowed down, the non-adiabatic Aharonov-Anandan (AA) phase^13^, the area enclosed by the quantum trajectory goes to the adiabatic Berry phase, the area enclosed by the adiabatic path. However, the time-energy uncertainty, the length of the quantum trajectory, *does not* converge to the minimum time-energy uncertainty of the adiabatic path. This singular feature of the adiabatic approximation is explained by the arc length and arc area of the cycloid. In addition, the cycloid curve neatly explains some interesting physical results. First, the axis trajectory of a cycloid is interpreted as a transitionless driving that makes the quantum evolution follow the adiabatic path. Second, the non-adiabatic resonance condition is visualized by the number of perfect arcs of the cycloid. Finally, the exact cycloid, curtate and prolate cycloids on a Bloch sphere are generated by different initial states. Our results could be tested with a single qubit, a neutron, or light polarization, and could have important implications for the application of the adiabatic perturbation, for example, adiabatic quantum computing and adiabatic quantum dynamics.

## Results

### Spin-1/2 particle in a rotating magnetic field

We consider one of the simplest quantum systems, a spin-1/2 particle in a rotating magnetic field 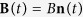 where we assume its strength *B* is constant and its direction **n**(*t*) rotates with constant angular speed *ω*. Quantum dynamics is governed by the time-dependent Schrödinger equation


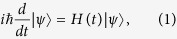


where the time-dependent Hamiltonian is given by the Zeeman interaction


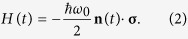


Here *ω*_0_ is the Larmor frequency (for the electron spin: 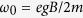), and ***σ*** is the Pauli spin vector. As Feynman *et al*.^14^ showed, with a Bloch vector 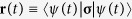 Equation (1) can be written as the dynamics of a spinning top


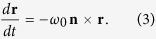


Before discussing the solution of Equation (1), let us recall the adiabatic dynamics of a spin-1/2 particle. The adiabatic theorem states that when an applied magnetic field changes slowly enough, a quantum state 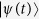 adiabatically evolving from an initial instantaneous state 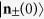, remains in an instantaneous eigenstate 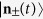 up to the dynamical phase 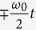 and the adiabatic geometric phase *γ*_±_ (called the Berry phase)^11^,^12^


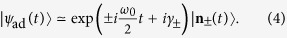


The instantaneous eigenstates 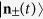 are the solution of 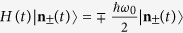 and written as 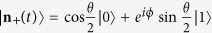 and 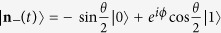. Here *θ* and *ϕ* are the polar and azimuthal angles of 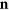, respectively. In the adiabatic limit, the Bloch vector 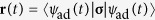, i.e., the spin direction is aligned with the direction of the magnetic field **n**(*t*) in the parameter space. The Berry phase is expressed in terms of the geometric quantity, the solid angle ***S*** subtended by **n**(*t*) as 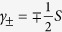.

The question we would like to explore is how the non-adiabatic trajectory of the Bloch vector **r** approaches that of **n** when the magnetic field rotates slowly. To this end, the exact solution of Eq. (1) is obtained by transforming the equation into the adiabatic frame via the transformation 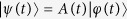, where *A*(*t*) is composed of the column vectors 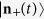 and 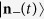. In the adiabatic frame, the time-dependent Schrödinger Eq. becomes


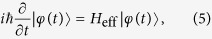


where the effective Hamiltonian is decomposed into the sum of the adiabatic and the non-adiabatic terms


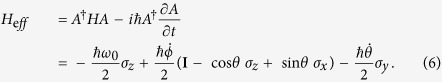


For the magnetic field rotating with constant angular speed, the effective Hamiltonian (6) becomes time-independent and Eq. (1) is exactly solvable.

We consider two cases: (i) a rotation along the latitude, 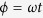 and constant *θ*, and (ii) a rotation along the meridian, 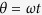 and constant 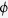. In the adiabatic frame, the Bloch vector **r**(*t*) rotates with the frequency Ω around the new axis 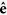 at an acute deviated angle *α* relative to the 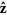 axis (adiabatic axis). The frequency Ω and the deviated angle *α* are given by 
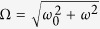
 and 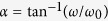 for the rotation along the meridian, and 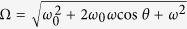 and 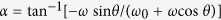 for the rotation along the latitude, respectively. The slowness of the rotation of the magnetic field is relative to the Larmor frequency. So the ratio of two frequencies, 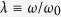, controls the adiabaticity of the quantum dynamics. In the limit of 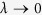, the quantum dynamics becomes adiabatic, and the radius *a* of the (imaginary) rolling circle along the adiabatic path, given by 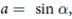 becomes smaller. The exact solution to Eq. (1) is written as


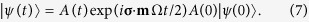


### Cycloids on a Bloch sphere

**Figure 1:**
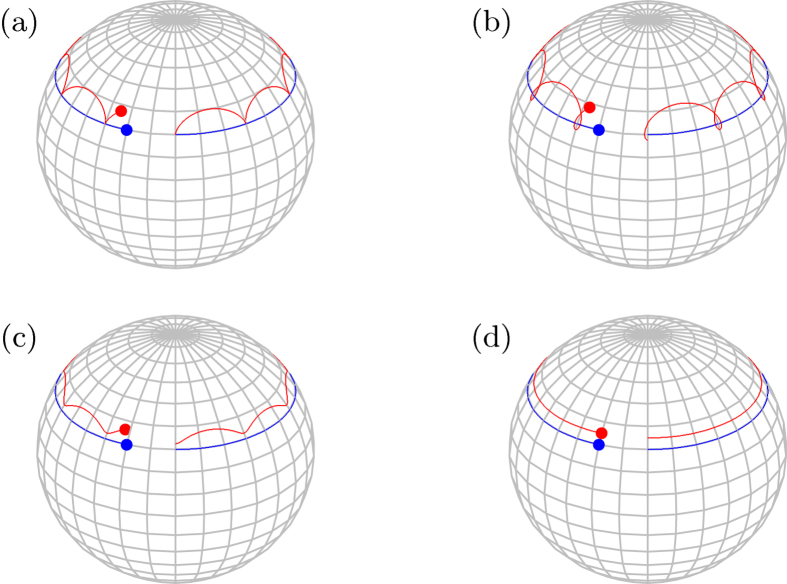
Cycloids on a Bloch sphere. As the rotating magnetic field traces the blue line, the Bloch vector makes various trajectories in red: (**a**) the exact cycloid, (**b**) prolate cycloid, (**c**) curtate cycloid, and (**d**) the axis trajectory, depending on the initial conditions.

We calculate the trajectory of the Bloch vector **r**(*t*) on the Bloch sphere with the exact solution Eq. (7). Figure 1 plots the cycloids traced by the Bloch vector 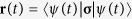 on a Bloch sphere for different initial states when the magnetic field is rotated clockwise with angular speed 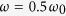 around the *z*-axis by the azimuthal angle 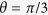. On a plane, a cycloid is the curve traced by a point on the rim of a circle that rolls along a straight line. In classical mechanics, it is the solution to two famous problems: brachistochrone (shortest-time) curves and tautochrone (equal-time) curves^15^,^16^. Like on a plane, a cycloid on the Bloch sphere is traced by a point of an imaginary circle which rolls along the base line. Here the adiabatic path **n**(*t*), i.e., the trajectory of the magnetic field, plays the role of the base line, as shown by the blue curve in Fig. 1. The imaginary circle rolling along the adiabatic path represents a non-adiabatic quantum dynamics. The radius of the rolling circle is determined by two frequencies *ω*_0_ and *ω*, and given by 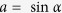. The slower the rotation of the magnetic field, the smaller the rolling circle becomes. This clearly illustrates how the quantum trajectory approaches the adiabatic path in the adiabatic limit.

Like a cycloid on a plane, in addition to the instantaneous eigenstate of the initial Hamiltonian, any initial state corresponding to a point on the rim of the rolling circle generates a cycloid. Initial states inside and outside of the rolling circle trace a curtate cycloid and a prolate cycloid on the Bloch sphere, respectively. The arc length and arc area of the cycloid on the sphere were obtained by Bjelica^17^. Table 1 shows the comparison of cycloids on a plane and on a sphere. As shown by the blue curve in Fig. 1, the curve traced by the axis of the rolling circle is parallel to the base line, i.e., the adiabatic path. The axis path can be interpreted as an example of transitionless driving^18^,^19^,^20^ to accelerate the adiabatic evolution. This could be understood by considering the axis path as a new evolution path of the spin, which is driven non-adiabatically by the parallel-rotating magnetic field, not by a slowly rotating magnetic field along the axis path. More specifically, this can be done by adding a transitionless-driving Hamiltonian *H*_*D*_(*t*) to cancel the non-adiabatic effect of the original time-dependent Hamiltonian *H*(*t*). As shown in Eq. (6), the original Hamiltonian *H*(*t*) is transformed to 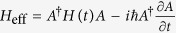 in the adiabatic frame. Here the first term is diagonal with respect to the instantaneous eigenstates of *H*(*t*), and the second term is off-diagonal and accounts for the non-adiabatic effect. A Hamiltonian 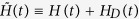 with a transitionless-driving term 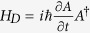 is transformed to the diagonal Hamiltonian 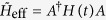 in the adiabatic frame. So if a Hamiltonian 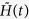 is applied, a quantum state initially in an instantaneous eigenstate of *H*(0) remains in the instantaneous eigenstate of *H*(*t*), and a non-adiabatic evolution along the adiabatic path of *H*(*t*) is achieved. This technique could be used to flip neutron spins non-adiabatically.

### Non-adiabatic resonance

The non-adiabatic term, the second part in Hamiltonian (6), causes the quantum trajectory to deviate from the adiabatic path. The question we address now is how the quantum evolution follows the adiabatic path as the rotation of the magnetic field becomes slower. Even before approaching the adiabatic limit, an evolved state could end up in the adiabatic target state under some condition. This is the so-called adiabatic resonance. Let us consider the magnetic field rotated by an angle *β* during the time *T*, i.e., 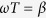. Clearly, the Bloch vector **r**(*t*), which is initially aligned to the *z*-axis in the adiabatic frame (that is, the instantaneous eigenstate at *t* = 0), will point again to the *z*-axis if the Bloch vector in the adiabatic frame rotates precisely *n* times, i.e., 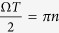. The non-adiabatic resonance condition is thus given by


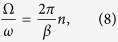


where *n* is the number of cycloid arcs.

### Arc area of a cycloid as geometric phase

Let us take a close look at the physics associated with the geometric properties of a cycloid curve on a Bloch sphere. The length and area are two basic geometric quantities of a curve. First, we discuss the area enclosed by the cycloid curve. To this end, we consider again the magnetic field which rotates completely around the *z*-axis by the azimuthal angle *θ* as shown in Fig. 1. As is well known, when the magnetic field rotates slowly enough, the evolved state remains in the instantaneous eigenstate, Eq. (4), and accumulates, in addition to the dynamical phase, a Berry phase which is proportional to the solid angle enclosed by the adiabatic path **n**(*t*)^11^. When the adiabatic condition is relaxed, a quantum state accumulates both dynamical and geometric phases. The latter is called the Aharonov-Anandan (AA) phase, which is proportional to the area of the curve of the quantum evolution in the projected Hilbert space^13^. When the resonance condition is satisfied, the Bloch vector **r**(*t*) returns to its initial position after a complete rotation of the magnetic field, so that the cycloid curve is closed. This allows us to calculate the AA phase and explore how the AA phase approaches the Berry phase in the adiabatic limit.

The AA phase *γ*_*AA*_^13^ is defined by subtracting the dynamical phase *γ*_*d*_ from the total phase *γ*_*t*_


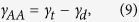


where the dynamical phase is defined by


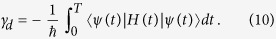


When the magnetic field rotates completely around the *z* axis for the time 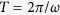, the quantum state also returns to the initial state 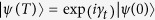 up to the total phase *γ*_*d*_ if 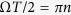, with 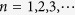. Here *n* represents the number of complete cycloid arcs. From 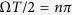 and 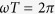, the ratio of the two frequencies to make the cycloid closed is given by 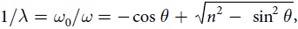 for 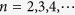. For *n* = 1, one has 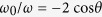. With the exact solution (7), one obtains 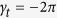 for odd *n* or 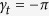 for even *n*, up to 2*πk* (*k* = integer). The dynamical phase, Eq. (10), is calculated as


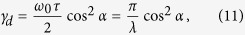


where 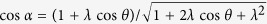 and 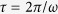. As expected, the dynamical phase becomes 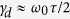 in the adiabatic limit because 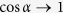 as 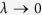. The AA phase up to 2*πk* (*k* = integer) can be written as


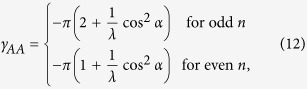


where the integer *n* is defined by the relation 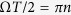.

In the adiabatic limit, i.e., 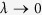, one may think that *γ*_*AA*_ would diverge because of the second term 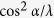 of Eq. (12). However, the phase is defined up to 2*πk*. The condition 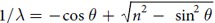 becomes 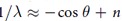, for 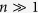. Thus, in the adiabatic limit, the AA phase, Eq. (12) converges to the Berry phase


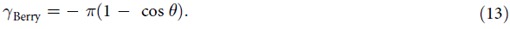


Note that the above equation holds for both even and odd *n*. As shown in Fig. 1, the difference between AA and Berry phases is the sum of the arc areas of a cycloid. In the adiabatic limit, i.e., 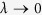, the arc area of a cycloid on a sphere becomes the arc area on a plane,


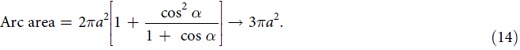


When the magnetic field is rotated around the *z*-axis by the angle *θ*, the total number *n* of arc areas is calculated from the resonance condition: 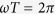 and 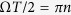, i.e., 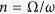. The radius of a cycloid is written as 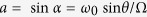. Thus the total arc area in the adiabatic approximation is given by


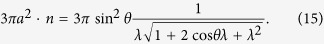


This is inversely proportional to *λ* so that the difference between the AA and Berry phases vanishes as 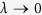. That is, the AA phase becomes the Berry phase in the adiabatic limit, as expected.

### Length of a cycloid as time-energy uncertainty and its singular limit

Let us turn to the physics related with the length of the cycloid curve. The Heisenberg position-momentum uncertainty relation is based on the commutation relation between two operators. However, the energy-time uncertainty is different because time in quantum mechanics is not an operator. Anandan and Aharonov^21^ gave a nice interpretation of the energy-time uncertainty relation as the distance of the quantum evolution measured by the Fubini-Study metric in the projective Hilbert space. The length *L* of the quantum evolution between two orthogonal states, here from 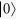 to 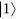, is expressed as


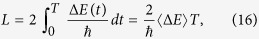


where 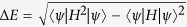 is the uncertainty in the energy during the running time *T*. The length of any curve connecting two orthogonal states 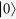 and 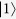 is greater than or equal to the shortest distance between them, i.e., the geodesic line of length *π*, 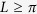. So the energy-time uncertainty is given by


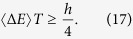


In other words, the minimum time *T* required for transforming a quantum state to an orthogonal one is bounded by 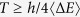, as shown by Mandelstam and Tamm^22^, Fleming^23^, Vaidman^24^, and Levitin and Toffoli^25^.

**Figure 2:**
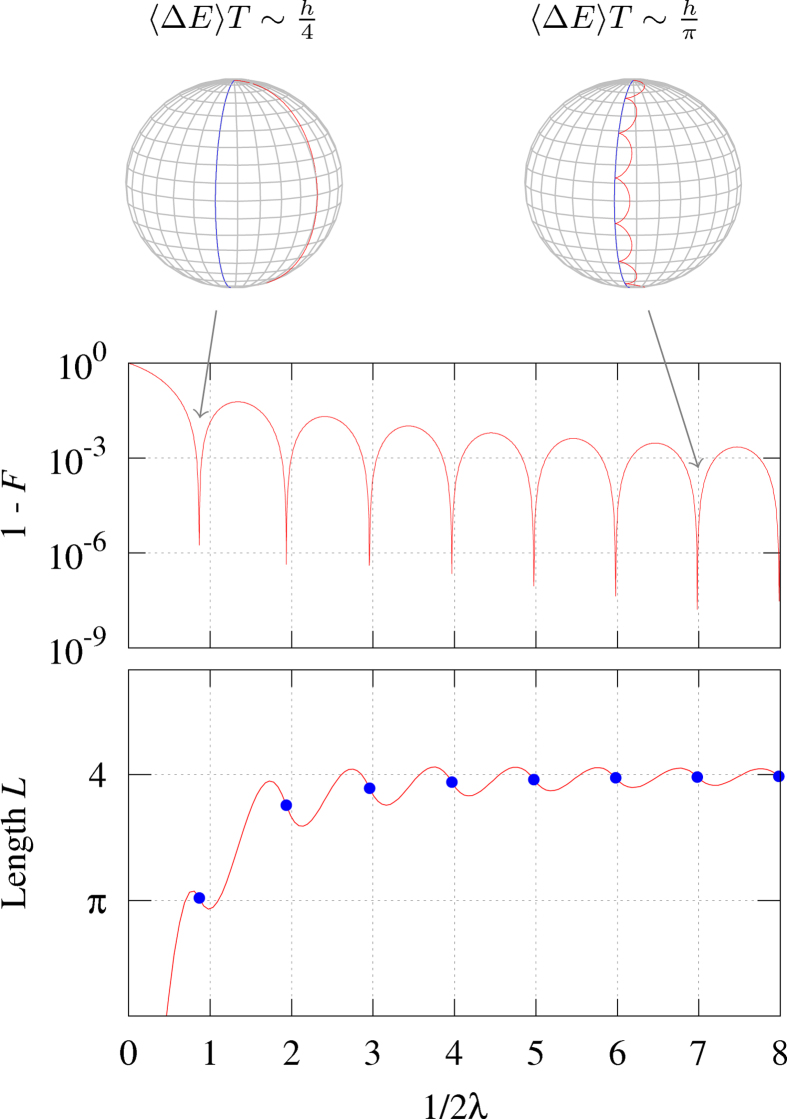
Trajectories, infidelity, and length. The top panel shows two trajectories, in red, on a Bloch sphere for 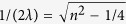 with *n* = 1 and 7, respectively. Here *n* represents the number of complete cycloid arcs. The blue line is the adiabatic path or the trajectory of a magnetic field. The middle and bottom panels plot the infidelity, the probability deviating from 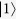, and the distance of the quantum evolution, respectively, as a function of the adiabatic parameter *λ*. The blue points in the bottom panel represent the perfect transition.

Now let us examine how the length of the quantum evolution changes as the speed of a rotating magnetic field from the north to the south pole along the geodesic line varies, as shown in Fig. 2. The adiabatic theorem dictates that if the magnetic field rotates slowly enough, the quantum evolution is well approximated by an instantaneous eigenstate. As depicted in Fig. 2, in the adiabatic limit, the path of the quantum evolution approaches the adiabatic path, i.e., the geodesic line. One would expect that the length of the quantum evolution in the adiabatic limit becomes just that of the adiabatic path (the geodesic line) because the difference between the two paths, measured by the enclosed area (difference between the AA and Berry phases) vanishes. So the time-energy uncertainty in the adiabatic limit would be minimum. However, this is not the case. In the adiabatic limit the length of the quantum evolution becomes *L* = 4, not *π*, as explained below. This is called the diagonal paradox or the limit paradox^26^ in calculus. Some well-known examples showing singular limits^27^ are the classical limit of quantum mechanics, and the limit of zero viscosity^8^ called d’Alembert’s paradox.

With the exact solution of the Schrödinger equation, one can calculate the length of the quantum evolution. After some algebra, the length *L* as a function of adiabatic parameter *λ* is given by


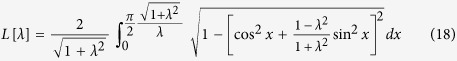


In the limit 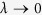, while the integrand of Eq. (18) becomes smaller, the interval of integration become larger. So one obtains


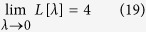


which is greater than the geodesic length *π* between the north and the south poles. This result can be also understood in terms of the product of the arc length of a cycloid and the number of cycloid arcs needed. At the non-adiabatic resonance condition with 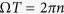 and 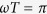, the radius *a* of the cycloid is given by 
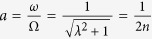
. In the adiabatic limit, the cycloid can be seen as a plane cycloid, so the arc length is just 8*a*. Thus the length becomes 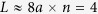.

Figure 2 plots the infidelity, the probability of deviating from the target state 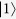, and the distance *L* of quantum evolution as a function of the adiabatic parameter 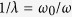. At the first non-adiabatic resonance, i.e., 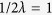, the curve is composed of a single segment of a cycloid and deviates from the adiabatic path. However, its length is slightly larger than *π*. This implies 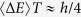. On the other hand, in the adiabatic limit, i.e., 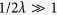, the curve is composed of many cycloids with smaller radius, which gets close to the adiabatic path with length *π*. In the adiabatic limit of 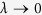, while the red curve in Fig. 2 approaches the blue one, the adiabatic path, its length converges to 4 not to *π*, as shown before. The time-energy uncertainty becomes 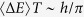, which is not minimum. Recall that within the quantum adiabatic theorem, the instantaneous eigenstate (4) is a good approximation to the true quantum evolution if the Hamiltonian changes slowly enough. What we find here is different. While the non-adiabatic geometric phase indeed converges to the adiabatic geometric phase, the time-energy uncertainty does not. In other words, *the adiabatic limit is singular* in the sense that the instantaneous eigenstate cannot capture all the physical properties in this limit.

## Discussion

In conclusion, we have shown that the Bloch vector of a spin in a rotating magnetic field traces a cycloid on a Bloch sphere. Like on a plane, different initial states trace prolate and curtate cycloids, and a trajectory parallel to the adiabatic path. The perfect non-adiabatic resonance is geometrically interpreted as the complete rolling of a cycloid. Two fundamental geometric quantities, the area enclosed by a cycloid curve and its length, are connected to two physical quantities, the geometric phase and the time-energy uncertainty, respectively. The arch areas of a cycloid give rise to the difference between the AA phase and the Berry phase. The energy-time uncertainty becomes 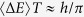 which is greater than the minimum time-energy uncertainty *h*/4. We found the quantum adiabatic limit is singular, similar to the diagonal paradox or d’Alembert’s paradox in the limit of zero viscosity. In the adiabatic limit, while the AA phase converges to the Berry phase, the length, time-energy uncertainty, does not converge to that of the adiabatic path. In other words, as we approach the adiabatic limit, some non-adiabatic errors integrated over the longer duration of the adiabatic cycle do not vanish^28^.

In mathematics, the isoperimetric inequality gives the relation between the circumference *L* of a closed curve and the area *A* it encloses. The isoperimetric inequality on a sphere^29^ is given by


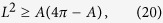


where the equality holds if and only if the curve is a circle. In our case, this inequality gives the relation between the geometric phase and the time-energy uncertainty. For a slow rotation of the magnetic field along the great circle passing from the north to the south pole, the area is given by *A* = 2*π*, and the quantum state acquires the phase factor −1. If the quantum trajectory were the great circle, its length would be *L* = 2*π*, according to the equality condition of the isoperimetric inequality. The actual distance of the quantum evolution is 8, not 2*π*, even though it looks like a great circle.

The geometric phases, the area enclosed by a cycloid curve, have been measured with various spin-1/2 systems such as a neutron in a rotating magnetic field, the polarization of light in a coiled optical fiber, and qubits. The time-energy uncertainty, the length of a cycloid curve, and its singular limit can be measured with these systems as well. While the geometric phase is measured via interference between an evolved and initial quantum states, a trajectory on a Bloch sphere seems necessary for calculating the time-energy uncertainty because of difficulty in measuring energy fluctuation. However, with the rapid advancement in manipulating qubits, it is possible to track a trajectory of a qubit on a Bloch sphere. Especially, we notice that Roushan *et al*.^30^ traced the cycloid curve on a Bloch sphere in an experiment measuring the non-adiabatic geometric phase with superconducting qubits.

The adiabatic approximation is one of the fundamental theorems in quantum mechanics, and has many applications; for example, the Born-Oppenheimer approximation and adiabatic quantum computing. The results here could give an opportunity to deepen our understanding of the adiabatic approximation.

## Methods

### Time-evolution of a spin in a rotating magnetic field

The evolved quantum state at time *t* is given by


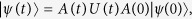


where *U*(*t*) is the time-evolution operator in the adiabatic frame. Here the operator *A*(*t*) has 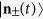 as its column vectors and is given explicitly by


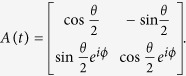


For a magnetic field rotating about the *z* axis by an angle *θ*, the effective Hamiltonian in the adiabatic frame is given by


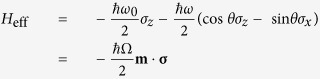


where 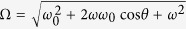, 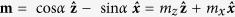 is the direction of the effective magnetic field in the adiabatic frame, 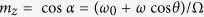, and 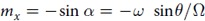. Thus, the time-evolution in the adiabatic frame is given by 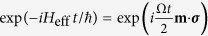.

### Calculation of the time-energy uncertainty

The magnetic field is rotated from the north pole to the south pole along the geodesic line. The initial state is 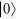. With the exact solution, it is straightforward to calculate the length 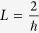 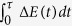


Where


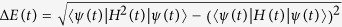
.

One obtains


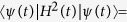
 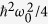


and


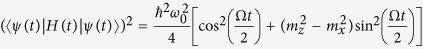


where 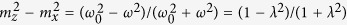. By changing the variable in the integrand, one obtains Equation (18).

### Calculation of the trajectory

The trajectory of the Bloch vector 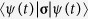 is plotted with the exact solution and by solving the time-dependent Schrödinger equation numerically with the Runge-Kutta method.

## Additional Information

**How to cite this article**: Oh, S. *et al*. Singularity of the time-energy uncertainty in adiabatic perturbation and cycloids on a Bloch sphere. *Sci. Rep*. **6**, 20824; doi: 10.1038/srep20824 (2016).

## Figures and Tables

**Table 1:** Comparison between cycloids on a plane and on a sphere.

	plane cycloid	spherical cycloid
base line	straight line	adiabatic path **n**(*t*)
circle radius	*a*	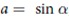
rolling speed	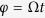	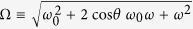
equations	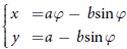	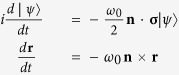
curtate/prolate	in/outside	in/outside
arc length	8*a*	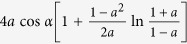
arc area	3*πa*^2^	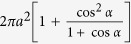

In plane case, the curtate and prolate cycloids are traced by a point at radii *b* < *a* and *a* >*b*, respectively.
